# From Cell Death to Regeneration: Rebuilding After Injury

**DOI:** 10.3389/fcell.2021.655048

**Published:** 2021-03-18

**Authors:** Dylan J. Guerin, Cindy X. Kha, Kelly Ai-Sun Tseng

**Affiliations:** School of Life Sciences, University of Nevada, Las Vegas, Las Vegas, NV, United States

**Keywords:** regeneration, apoptosis, tissue repair, caspase, cell death, Wnt, BMP, liver

## Abstract

The ability to regrow lost or damaged tissues is widespread, but highly variable among animals. Understanding this variation remains a challenge in regeneration biology. Numerous studies from *Hydra* to mouse have shown that apoptosis acts as a potent and necessary mechanism in regeneration. Much is known about the involvement of apoptosis during normal development in regulating the number and type of cells in the body. In the context of regeneration, apoptosis also regulates cell number and proliferation in tissue remodeling. Apoptosis acts both early in the process to stimulate regeneration and later to regulate regenerative patterning. Multiple studies indicate that apoptosis acts as a signal to stimulate proliferation within the regenerative tissues, producing the cells needed for full regeneration. The conservation of apoptosis as a regenerative mechanism demonstrated across species highlights its importance and motivates the continued investigation of this important facet of programmed cell death. This review summarizes what is known about the roles of apoptosis during regeneration, and compares regenerative apoptosis with the mechanisms and function of apoptosis in development. Defining the complexity of regenerative apoptosis will contribute to new knowledge and perspectives for understanding mechanisms of apoptosis induction and regulation.

## Introduction

The ability to regenerate lost or damaged tissues is an impressive ability that is not common to all animals. How this feat is achieved by those that can is an ongoing question. Helpfully, a number of species with regenerative capacity are available as model organisms. Some invertebrates, such as the cnidarian *Hydra* and the planarian flatworm, display remarkable regenerative capacity. Both animals regenerate whole organisms from small body fragments (Trembley, 1744; Pallas, 1766). Hydra can even regenerate its entire body plan from reaggregated cells (Noda, 1971; Gierer et al., 1972). This regenerative capacity depends on reserves of active adult stem cells: pluripotent stem cells (neoblasts) in planaria and multipotent interstitial stem cells in *Hydra* (Wagner et al., 2011; Hobmayer et al., 2012; Scimone et al., 2014). Successful regeneration depends on these stem cells to respond to injury by proliferating and their progeny differentiating to return the organism to full structural and functional integrity (Baguñà, 1976; Saló and Baguñà, 1984; Reddien and Sanchez Alvarado, 2004).

Although vertebrates cannot regenerate an entire animal from small body fragments, some are remarkable for their ability to regrow substantial and complex body parts. One well-studied model is the zebrafish, *Danio rerio*, which regenerates several structures including the epidermis, retina, heart, and appendages (Marques et al., 2019). Another model with high regenerative capacity is the axolotl *Ambystoma mexicanum*, which can regenerate severed limbs and damaged hearts (Joven et al., 2019). Furthermore, some amphibians, such as the clawed frog *Xenopus laevis*, display age-dependent regeneration of larval tails, limbs, and embryonic eyes (Dent, 1962; Morgan and Davis, 1902; Beck et al., 2003; Kha and Tseng, 2018; Kha et al., 2018). This age-dependent feature of *Xenopus* facilitates an examination of the mechanisms that regulate endogenous changes in regenerative capacity and to test strategies for stimulating regeneration in non-regenerative states. Even though mammalian regenerative capacity is more restricted than in these other models, mammals can regenerate some tissues, including the liver and digit tips in mice and humans (Higgins and Anderson, 1931; Illingworth, 1974; Han et al., 2008), and the entire epidermis in the African spiny mouse (Seifert et al., 2012).

Across species and tissues, the broad steps of regeneration after injury are as follows: a successful wound healing response, the initiation of regeneration, followed by cell proliferation and cellular differentiation to rebuild lost tissues (reviewed in Gurtner et al., 2008; Pfefferli and Jaźwińska, 2015; Kakebeen and Wills, 2019). The source of the contributing cell population varies between systems. Unlike invertebrates, vertebrate regeneration appears to often be achieved through the use of lineage-restricted progenitor cells, such as for limb regeneration in zebrafish (Poss et al., 2000; Stewart and Stankunas, 2012), axolotl (Kragl et al., 2009; Makanae et al., 2014), and *Xenopus* (Gargioli and Slack, 2004). Other mechanisms such as transdifferentiation to regenerate amphibian lens are also used (Eguchi, 1963; Freeman, 1963). The proliferation and differentiation of these cells to regenerate the lost organ requires the action of complex mechanisms, only some of which have been characterized.

One important mechanism in regeneration is apoptosis. Apoptosis, a type of programmed cell death, is a fundamental and evolutionarily conserved process (Metzstein et al., 1998). Apoptosis is required for organogenesis, tissue remodeling, homeostasis, wound healing, and regeneration (Elmore, 2007; Li et al., 2010; D’Arcy, 2019). Dysregulation of apoptosis can have severe consequences including cancer and autoimmune disorders (Goldar et al., 2015). Apoptosis is initiated by the cleavage of inactive initiator caspase proteins to expose their catalytic domains, which allow them to activate executioner caspases through subsequent cleavage events (Cohen, 1997). The executioner caspases then initiate a cascade of events resulting in the breakup of the cell into smaller apoptotic bodies. These are engulfed by macrophages, completing the process (Budai et al., 2019).

Apoptosis has been widely studied in many contexts and in diverse organisms (reviewed in Brill et al., 1999; Pérez-Garijo and Steller, 2015; Tuzlak et al., 2016). Apoptotic cells have been shown to exert diverse non-autonomous effects on neighboring cells through the release of mitogenic factors, inducing cell proliferation (Morata et al., 2011). The role of apoptosis as a regenerative mechanism was more recently identified. Prior work determined that apoptosis is required for regeneration across multiple organisms and tissues (Tseng et al., 2007; Chera et al., 2009; Li et al., 2010; Sîrbulescu and Zupanc, 2010; Gauron et al., 2013; Kha et al., 2018; Brock et al., 2019). How apoptosis promotes successful regeneration is beginning to be understood. In this review, we discuss the requirement of apoptosis in different regenerative contexts, the initiators and downstream effects of apoptosis during regeneration, and gaps in the field. We focus on recent advances that highlight the importance of apoptosis as a specific response to stimulate and regulate regeneration and not merely as a consequence of tissue damage.

## Initiation and Regulation of Regenerative Apoptosis

Apoptosis is commonly observed starting early in the regeneration process. Programmed cell death has long been known to contribute to wound healing after injury (Greenhalgh, 1998). However, apoptosis has additional and separable functions specific to regeneration after the initial wound healing phase. Pathways regulating these apoptotic events have been identified in some models.

Apoptosis occurs during the early phases of regeneration in two peaks in some regenerative systems. In *Hydra* and planaria, the first peak of apoptosis occurs from 1 to 4 h after bisection (Chera et al., 2009; Beane et al., 2013). At 3 days, there is a second peak of apoptosis (Pellettieri et al., 2010). A similar pair of apoptotic peaks is seen during adult zebrafish fin regeneration from 1 to 12 h post amputation (hpa) and 15 to 72 hpa. The second peak of apoptosis is specific to regeneration, as simple wounding of the fin that healed quickly failed to induce this second peak (Gauron et al., 2013). In *Xenopus laevis* tadpole tail regeneration, there is only one sustained increased in apoptosis at the injury site. Apoptosis is absent during the wound healing phase and is first activated during formation of the regeneration bud at 12 hpa and remains active during the entire initial proliferative phase from 12 to 48 hpa (Tseng et al., 2007).

An important regulator of apoptosis in regeneration is BMP signaling (Guimond et al., 2010). BMP signaling regulates anterior-posterior patterning, proliferation, differentiation, and apoptosis in the developing vertebrate limb (Pignatti et al., 2014). Here, BMP activates apoptosis in the apical ectodermal ridge and the limb mesenchyme (Gañan et al., 1998; Guha et al., 2002). BMP signaling is also required for regeneration of axolotl limbs (Guimond et al., 2010; Vincent et al., 2020). In this system, the regulation of apoptosis by BMP2 appears to function the same for both the developing and regenerating limb (Guimond et al., 2010). The overexpression of BMP2 increased apoptosis in the regenerating limb, while overexpression of the BMP inhibitor Noggin caused a decrease in apoptosis relative to controls (Guimond et al., 2010). A similar mechanism is also seen in mouse digit formation, where inhibition of BMP via Noggin caused a reduction in apoptosis in the inter-digit region, resulting in a flipper like appendage instead of a hand (Wang et al., 2004).

Another well-known signaling pathway active in apoptosis induction during regeneration is the Jun-N terminal Kinase (JNK) signaling pathway (Santabárbara-Ruiz et al., 2015; Diaz-Garcia et al., 2016; Camilleri-Robles et al., 2019). JNK regulates apoptosis in the developing brain. A knockout of *Jnk1* and *Jnk2* caused both reduced apoptosis in the hindbrain and increased apoptosis in the forebrain (Kuan et al., 1999). JNK signaling is required for both apoptosis and regeneration following bisection in planaria (Almuedo-Castillo et al., 2014). In *Drosophila* wing imaginal disc regeneration, JNK signaling is required to induce apoptosis (Diaz-Garcia et al., 2016). Together, the studies suggest that JNK plays a regulatory role for apoptosis in development that may act similarly in regenerative apoptosis.

There is an interesting link between apoptosis and reactive oxygen species (ROS). ROS are detected early in regeneration and are necessary for regeneration in multiple species (Love et al., 2013; Ferreira et al., 2018; Novianti et al., 2019). Perhaps not coincidentally, ROS are important for apoptosis-dependent regeneration. In zebrafish fin regeneration, ROS levels increased at the injury site immediately following amputation and continued rising for 16 h before returning to baseline. The high levels of ROS around 15 hpa correlated closely with the beginning of the second round of apoptosis. Additionally, ROS levels peaked at 2 h after wounding injuries to the fin that did not require tissue regeneration, suggesting a specific importance of ROS for apoptosis-dependent regeneration (Gauron et al., 2013). ROS are also required for regeneration of the *Drosophila* imaginal disc, where they regulate JNK signaling (Santabárbara-Ruiz et al., 2015). The intersection of ROS, JNK signaling, and apoptosis is an exciting direction for further investigation.

## Regulation of Regenerative Mechanisms by Apoptosis

Apoptosis can promote proliferation during development, notably in the developing *Drosophila* imaginal discs where apoptotic cells promote compensatory proliferation in neighboring cells (reviewed in Diwanji and Bergmann, 2018). Although the specific roles of apoptosis in regeneration are still being explored, it is known that apoptosis can drive proliferation during regeneration in *Hydra*, planaria, *Xenopus*, and zebrafish. As proliferation is a critical aspect of regeneration (Gargioli and Slack, 2004; Jopling et al., 2010; Kha et al., 2018; Stocum, 2019), apoptosis is therefore an important regulator of regeneration.

Apoptotic cells can act as initiators of cell signaling in development (Morata et al., 2011). This is also true in regenerative contexts, for example the importance of apoptotic cells as a source of Wnt3 during *Hydra* head regeneration (Chera et al., 2009). Wnt/β-catenin signaling is also stimulated by apoptosis in zebrafish epithelium regeneration (Brock et al., 2019). In both systems, the Wnt ligand is found in apoptotic bodies and engulfed by neighboring stem cells to induce proliferation. This Wnt-induced proliferation is required for regeneration, as inhibition of Wnt signaling abolished regeneration (Chera et al., 2009; Brock et al., 2019). The observation of apoptosis-induced Wnt-dependent proliferation in both an invertebrate and a vertebrate is exciting, suggesting a potential conserved regenerative mechanism that could be tested as a strategy to induce mammalian regeneration.

Apoptotic cells secrete additional mitogenic signals in a number of models (Ryoo and Bergmann, 2012). Apoptotic cells in *Drosophila* secrete Hedgehog (the ortholog of vertebrate Sonic hedgehog) to induce proliferation during eye development (Fan and Bergmann, 2008). Prostaglandin signaling downstream of Caspase activity induces proliferation in mouse cells (Li et al., 2010), with a similar role proposed in zebrafish hematopoiesis (North et al., 2007). Whether apoptosis induces proliferation through these pathways during regeneration remains unclear.

Apoptosis as a patterning mechanism has been extensively studied in development (reviewed in Pérez-Garijo and Steller, 2015; Lin and Xu, 2019). However, much less is known about the patterning role of apoptosis in a regenerative context. An excellent example of an apoptosis requirement for pattering in regeneration is its asymmetric distribution and differential function following bisection in planaria and *Hydra*. Organismal bisection generates both anterior and posterior segments. Although each fragment undergoes apoptosis after bisection, the number of apoptotic cells is significantly higher in the head-regenerating posterior fragment than the tail-regenerating anterior fragment (Chera et al., 2009; Pellettieri et al., 2010). In *Hydra*, inhibition of apoptosis in the head-regenerating fragment blocked head regeneration. However, apoptotic inhibition of the foot-regenerating fragment did not block foot regeneration. Ectopic induction of apoptosis in the foot-regenerating fragment resulted in animals with heads in the presumptive foot region (Chera et al., 2009). These findings showed that a higher level of apoptosis is needed for head restoration, whereas a lower level is sufficient for induction of proliferation. In planarian regeneration, the second apoptotic peak is regulated by bioelectrical signaling and required for proper head patterning but not cell proliferation. Inhibition of the ion transporter H^+^, K^+^-ATPase caused a reduction of apoptosis and resulted in a shrunken head due to the lack of adjustment in organ size and placement (Beane et al., 2013). These studies indicate that apoptosis plays an instructive role in regenerative patterning of complex tissues.

## The Goldilocks Principle in Regenerative Apoptosis

Apoptosis is a potent mechanism that needs to be tightly controlled in regeneration. As in the tale of Goldilocks, the level of apoptosis needs to be “just the right amount” for successful regeneration. During regeneration, unchecked apoptosis could deplete the tissue of the cellular materials necessary to regenerate if it outpaces proliferation. Although one functional consequence of apoptosis is to promote proliferation, apoptosis itself is not always a marker of regeneration, even in normally regenerative tissues. For example, apoptosis is necessary for regeneration of the *Xenopus* tadpole tail. However, if the tail is amputated during the refractory period when the tadpole temporarily loses its tail regeneration ability, there is increased activated Caspase-3 activity relative to the regenerative tail (Tseng et al., 2007), suggesting that there is likely a limiting mechanism where a specific level of apoptosis is required for regeneration. Similarly, if the salamander limb is denervated during regeneration, the normally regenerative tissues will morphologically regress due to increased apoptosis (Mescher et al., 2000). During mouse liver regeneration, Nitric oxide synthase-deficient animals exhibit both increased apoptosis and decreased regeneration relative to controls (Rai et al., 1998).

IAPs (Inhibitors of apoptosis proteins) are known regulators of apoptosis in development and disease yet their role in regeneration is unclear. Bcl-2 orthologs are expressed during axolotl limb regeneration (Bucan et al., 2018). However, the expression patterns of IAPs in other models have not been defined. Overexpression of p35 (a baculoviral caspase inhibitor) showed only a minor effect on *Drosophila* wing disc regeneration (Diaz-Garcia et al., 2016). Similarly, Bcl-2 overexpression did not enhance axonal regeneration of retinal ganglion cells in mice (Inoue et al., 2002). Further molecular and functional studies are needed to assess the potential roles of IAPs in regulating regenerative apoptosis.

## Mammalian Regeneration: Apoptosis in the Regenerating Liver

Mammals, including humans, have limited regenerative ability but are able to regrow lost liver tissues following partial hepatectomy—resection of up to two thirds of the liver (Higgins and Anderson, 1931). Apoptosis in this context also differs somewhat from its conserved role in the models discussed in the earlier sections. An additional complexity in studying the role of apoptosis in liver regeneration is that different regenerative mechanisms can be activated depending on the method of injury (reviewed in Fausto et al., 2006; Mao et al., 2014). Following partial hepatectomy or acute chemical damage to the mammalian liver, TNF is released, stimulating ROS and NF-κB to induce apoptosis. Apoptosis induced neighboring progenitors to proliferate (Czaja et al., 1989; FitzGerald et al., 1995). Consistent with this finding, mice deficient in both Caspase-3 and Caspase-7 showed decreased liver progenitor proliferation, and impaired wound healing and regeneration following partial hepatectomy (Li et al., 2010). The liver is a distinct case from the earlier examples because mammalian liver regeneration results in the regrown liver achieving its former size but not its former shape. These may constitute distinct regenerative responses, which in turn may involve different roles for apoptosis.

## Discussion and Future Directions

**FIGURE 1:**
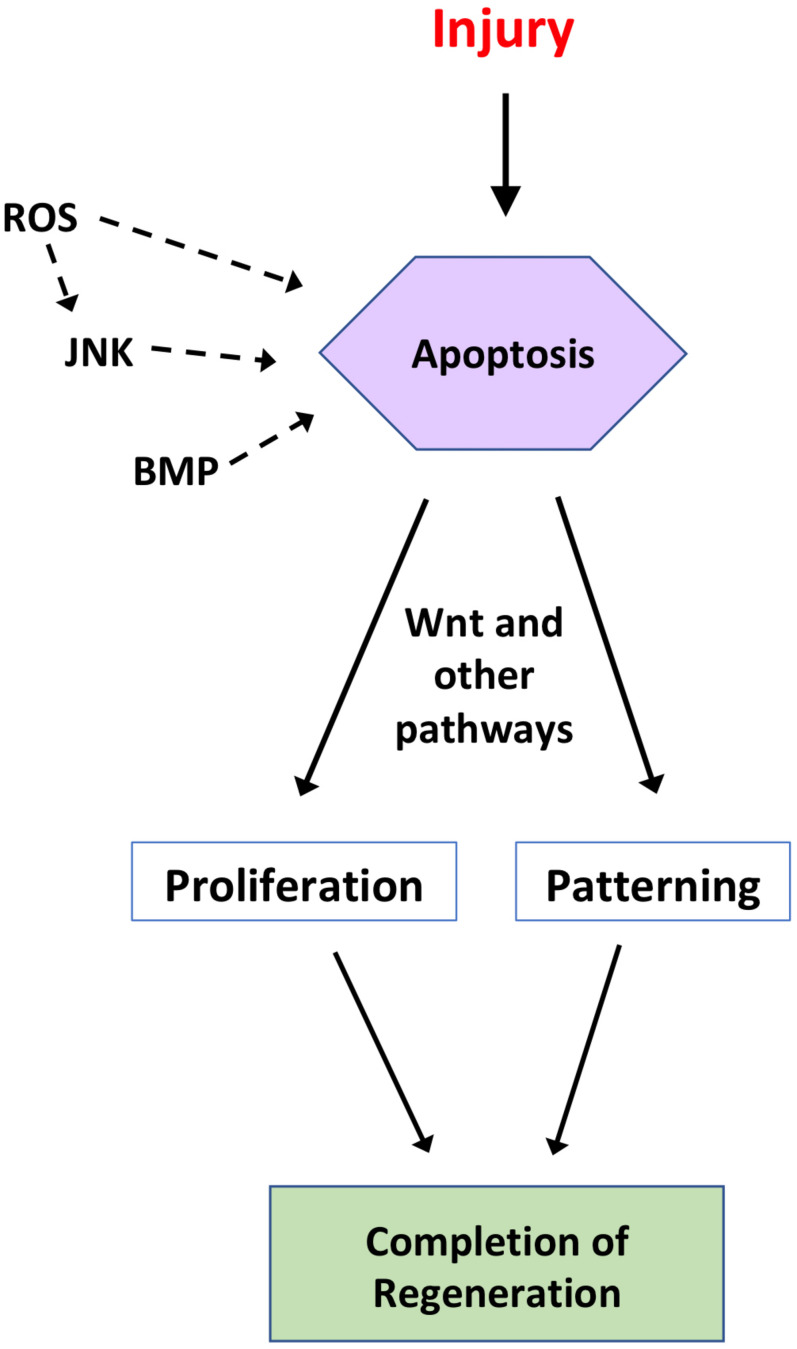
Summary of the regulation and roles of apoptosis during regeneration. Following injury to a tissue, apoptosis can act as a regenerative mechanism, depending on the organism and tissue in question. In some regenerative systems, apoptosis is regulated by ROS, JNK, and/or BMP signaling. In turn, apoptosis regulates proliferative and/or patterning signals, such as Wnt signaling, to drive initiation and completion of regeneration. This figure is not intended to be comprehensive and represents an amalgamation of studies from several model systems discussed in the text.

Apoptosis is a key mechanism of regeneration. Although apoptosis is demonstrably required for regeneration across diverse organisms and tissues, some important aspects of early stages of regeneration during which apoptosis is active are unknown. The roles of JNK, BMP and Wnt signaling pathways as inducers of apoptosis provide exciting leads but open questions remain (Figure 1). It is known that ROS are produced by macrophages following wounding (Bae et al., 2009). Moreover, the immune system is implicated in regulating endogenous regenerative ability (Fukazawa et al., 2009; reviewed in Mescher, 2017). A key aspect of the immune response is efferocytosis, the phagocytic clearance of apoptotic cells. In mice, reduced efferocytosis of apoptotic cardiomyocytes induced by myocardial infarction led to enlarged infarct size (Wan et al., 2013). In *Hydra* head regeneration, an immediate and large wave of efferocytosis by endodermal epithelial cells (immune-like cells) was seen beneath the injury plane (Chera et al., 2009) but whether this process plays an active role in regeneration is unknown. Regardless, these studies suggest that understanding the role of efferocytosis in regenerative apoptosis could be a promising area for investigation.

Another important question is which cell types are targeted for apoptosis and where they are located, which has been answered in only a few systems. Even among more fully characterized systems, such as *Hydra* head regeneration, only a portion of the interstitial stem cells undergo apoptosis, which leads to the question of whether there is a specific induction program or if it is a stochastic process. Additionally, in most systems, regenerative events must coordinate the outgrowth of multiple tissue types (Mochii et al., 2007; Lehoczky et al., 2011). Does each lineage undergo a separate round of apoptosis or is there one global apoptotic event that affects all lineages? One tool for addressing this question is the use of fluorescent reporters for caspase activation, allowing real time imaging of caspase activity (Bardet et al., 2008). Another powerful tool for answering this question is single-cell RNA sequencing, which allows for the analysis of individual cells during regeneration.

It is unknown whether regenerative apoptosis functions in the same manner across the multiple organisms and tissues in which it is found. WNT ligands found in apoptotic bodies during zebrafish epithelium and *Hydra* head regeneration act to promote proliferation (Chera et al., 2009; Brock et al., 2019). That this mechanism is conserved between an invertebrate and a vertebrate suggests that this potentially can be used to stimulate regeneration in non-capable tissues. Additionally, optogenetic tools can be used for spatiotemporal specificity in inducing apoptosis in the tissues of interest after injury (Jewhurst et al., 2014).

The mechanisms linking apoptosis to regeneration remain largely elusive. An intriguing finding is that apoptosis but not JNK signaling induced expression of a pluripotency marker during zebrafish fin regeneration, suggesting that regenerative apoptosis may influence cellular reprogramming (Gauron et al., 2013). Investigators may also turn to the regulation and functions of apoptosis in development for clues since developmental mechanisms are often used in regeneration (Beck et al., 2003; Lin and Slack, 2008; Taniguchi et al., 2014). However, the developmental role of apoptosis is not always recapitulated in regeneration. In zebrafish fin regeneration, JNK signaling induced proliferation but apoptosis induction is JNK independent (Gauron et al., 2013). In *Xenopus*, tail formation does not involve apoptosis, but apoptosis is required for tail regeneration (Johnston et al., 2005; Tseng et al., 2007). In this context, understanding how regenerative apoptosis is induced may provide strategies for stimulating regeneration in tissues where apoptosis does not normally play a developmental role.

The role of apoptosis in regeneration is an important one that merits a detailed investigation. Apoptosis can control the microenvironment in which tumors arise, making the study of apoptosis important for cancer treatment (Gregory et al., 2016). Similar to cancer, regeneration induces excessive cell proliferation—in this case, to restore a lost structure. In contrast to cancer, regenerative proliferation is tightly controlled such that the process terminates once the missing structure is restored. Comparative studies of apoptosis in cancer and regeneration may help to delineate the differences in controlled vs. dysregulated proliferation. Further investigations into this topic may provide new perspectives in understanding the functions of apoptosis in diseased tissues.

## Author Contributions

DG, CK, and KT contributed to the planning, drafting, and writing of the manuscript. All authors contributed to the article and approved the submitted version.

## Conflict of Interest

The authors declare that the research was conducted in the absence of any commercial or financial relationships that could be construed as a potential conflict of interest.
